# Apoptosis and Oxidative Stress in Human Intestinal Epithelial Caco-2 Cells Caused by Marine Phycotoxin Azaspiracid-2

**DOI:** 10.3390/toxins16090381

**Published:** 2024-08-31

**Authors:** Liye Zhao, Jiangbing Qiu, Jingrui Zhang, Aifeng Li, Guixiang Wang

**Affiliations:** 1 College of Environmental Science and Engineering, Ocean University of China, Qingdao 266100, China; zhaoliye6671@stu.ouc.edu.cn (L.Z.); zhangjingrui@stu.ouc.edu.cn (J.Z.); wangguixiang@stu.ouc.edu.cn (G.W.); 2 Key Laboratory of Marine Environment and Ecology, Ocean University of China, Ministry of Education, Qingdao 266100, China

**Keywords:** azaspiracids, Caco-2 cells, cell viability, oxidative damage, mitophagy

## Abstract

When humans consume seafood contaminated by lipophilic polyether phycotoxins, such as azaspiracids (AZAs), the toxins are mainly leached and absorbed in the small intestine, potentially causing intestinal damage. In this study, human intestinal epithelial Caco-2 cells were used to investigate the adverse effects of azaspiracid-2 (AZA-2) on human intestinal epithelial cells. Cell viability, apoptosis, oxidative damage and mitochondrial ultrastructure were investigated, and ribonucleic acid sequence (RNA-seq) analysis was applied to explore the potential mechanisms of AZA-2 toxicity to Caco-2 cells. Results showed that AZA-2 significantly reduced the proliferation of Caco-2 cells in a concentration-dependent response, and the 48 h EC_50_ of AZA-2 was 12.65 nmol L^−1^. AZA-2 can induce apoptosis in Caco-2 cells in a dose-dependent manner. Visible mitochondrial swelling, cristae disintegration, membrane rupture and autophagy were observed in Caco-2 cells exposed to AZA-2. Reactive oxygen species (ROS) and malondialdehyde (MDA) content were significantly increased in Caco-2 cells after 48 h of exposure to 1 and 10 nmol L^−1^ of AZA-2. Transcriptome analysis showed that KEGG pathways related to cellular oxidative damage and lipid metabolism were affected, mainly including mitophagy, oxidative phosphorylation, cholesterol metabolism, vitamin digestion and absorption, bile secretion and the peroxisome proliferator-activated receptor signaling pathway. The cytotoxic effects of AZA-2 on Caco-2 cells may be associated with ROS-mediated autophagy and apoptosis in mitochondrial cells. Results of this study improve understanding of the cytotoxicity and molecular mechanisms of AZA-2 on Caco-2 cells, which is significant for protecting human health.

## 1. Introduction

The group of azaspiracids (AZAs) is a class of polyether compounds with a spirocyclic structure, which can cause symptoms such as nausea, vomiting, diarrhea and stomach cramps in humans [1]. Some species of the genera of *Amphidoma* and *Azadinium* can produce AZAs, such as *Az. spinosum*, *Az. poporum* and *Am. languida* [2,3]. The *Azadinium* species have been found in the Mediterranean Sea, off the coast of Denmark, France, Ireland, Korea and China [3,4,5]. Currently, approximately 60 AZA analogues have been identified [6], and AZA-1, AZA-2 and AZA-3 are the most common compounds with high detection rates and toxicity [1]. These toxins are commonly accumulated in a variety of filter-feeding bivalve mollusks, such as mussels, scallops, oysters, cockles and clams [1,7]. Currently, AZA-2-producing microalgae are also widely distributed globally, and the amount of AZA-2 was generally in the range of a few femtograms per cell [2,3]. The analog AZA-2 was commonly accumulated in shellfish at concentrations ranging from 0 to 9.96 µg kg^−1^ [8,9,10]. Ingestion of shellfish contaminated by AZAs can lead to human poisoning events that have been reported in some regions of Europe [11]. To protect human health, the total content of AZA-1, AZA-2 and AZA-3 are regulated with a level of 160 μg AZA-1 equivalents kg^−1^ shellfish meat. Toxicological equivalence factors of AZA-1 = 1, AZA-2 = 1.8 and AZA-3 = 1.4 were adopted to convert individual AZAs to AZA-1 equivalents [1]. 

AZAs displayed toxic effects on cells in vitro, including decreases in cell viability, oxidative damage, apoptosis activation and modulation of the calcium inward flow pathway [12,13,14]. Moreover, AZA-1 can increase cellular caspase activity, induce irreversible cytoskeletal rearrangements, deplete cellular adenosine triphosphate, trigger cholesterol biosynthesis and inhibit bioelectrical activity, endocytosis of membrane proteins and intercellular adhesion [14,15,16,17]. Remarkable cytotoxic effects of AZA-1 have been observed in a variety of cellular systems, such as lymphocytes, primary neurons, Caco-2 cells and fibroblasts [1]. AZA-2 and AZA-3 may also induce DNA fragmentation in Caco-2 cells, increase cytosolic cyclic adenosine monophosphate levels in human lymphocytes and increase mitochondrial dehydrogenase activity in hepatocytes [13,18,19,20].

The human intestinal Caco-2 cell line possesses many characteristics of absorptive enterocytes in the small intestine [21] and can precisely simulate human intestinal epithelial cells, providing a valuable method for assessing the threat of phycotoxins to humans. The Caco-2 cell model has been applied to explore the toxicity and intestinal absorption of several phycotoxins on human intestinal epithelial Caco-2 cells [22]. Previous studies mainly focused on in vitro cytotoxicity studies of AZA-1 [23,24] and less on AZA-2 and other analogs [13]. AZA-2 is considerably more potent than AZA-1 and AZA-3 [1]. Furthermore, AZA-2 is a dominant profile of AZA-producing microalgae and is frequently detected in shellfish worldwide [2,4,5,25]. The main exposure to AZA-2 in humans is through the ingestion of seafood such as shellfish. After digestion of shellfish through the gastrointestinal tract, the leached toxin is mainly absorbed in the small intestine, potentially damaging intestinal cells. 

The toxicological effects of AZA-2 on human small intestinal epithelial cells are, however, not well understood. Therefore, Caco-2 cells were used as a model in this study, and the potential adverse effects of AZA-2 on Caco-2 cells, including cell viability, apoptosis, oxidative damage and mitochondrial ultrastructure, were investigated, and RNA-seq analysis was applied to explore the potential mechanisms of AZA-2-induced toxicity and apoptosis in Caco-2 cells. The results of this study will improve understanding of the cytotoxicity of AZA-2 and its potential hazard to human health.

## 2. Results

### 2.1. Effects of AZA-2 on the Viability and Apoptosis of Caco-2 Cells 

**Figure 1:**
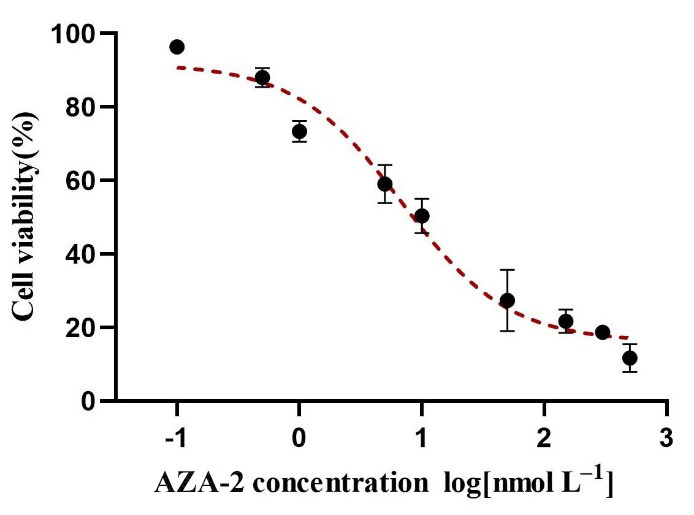
Viability of Caco-2 cells exposed to different concentrations of AZA-2 for 48 h. Data are shown as mean values ± standard error (n = 3).

The viability of Caco-2 cells after exposure to different concentrations of AZA-2 for 48 h is shown in Figure 1. After 48 h of exposure, AZA-2 significantly reduced Caco-2 cell proliferation (*p* < 0.05) with a concentration-dependent response. Cell viabilities were reduced by 12% and 90% after exposure to 0.5 and 500 nmol L^−1^ AZA-2, respectively. The 48-hour EC_50_ value of AZA-2 on Caco-2 cells was 12.65 nmol L^−1^.

**Figure 2:**
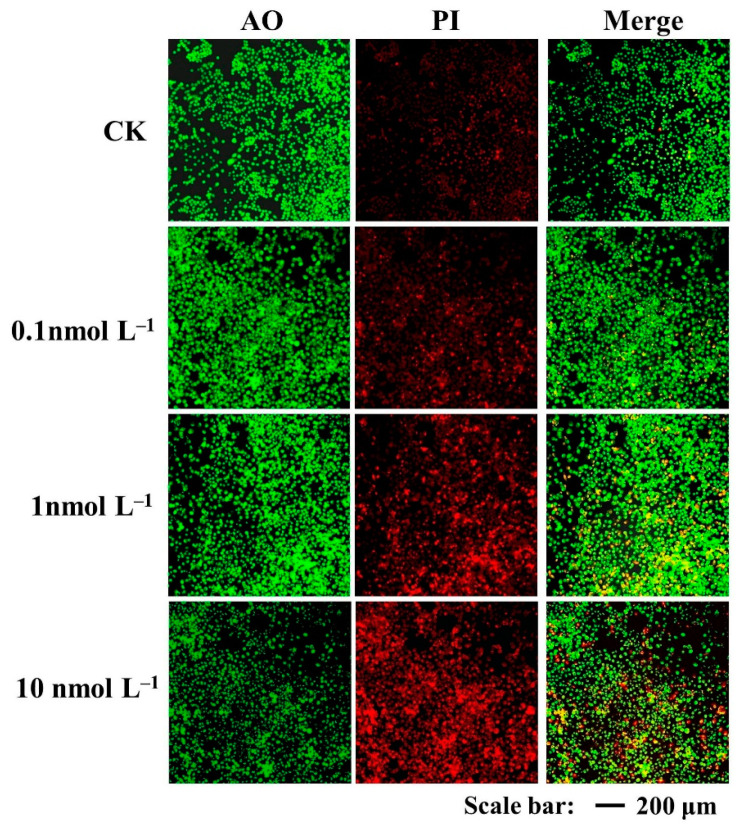
Fluorescence microscopy images of Caco-2 cells stained with AO/PI and exposed to various concentrations of AZA-2 for 48 h. (scale bar: 200 μm).

Apoptotic cells were detected by AO/PI staining with different fluorescent colors. With an increase in AZA-2 exposure concentration, green fluorescence was weakened and red fluorescence was enhanced for Caco-2 cells, indicating an increase in apoptotic and necrotic cells (Figure 2). Red fluorescence for Caco-2 cells exposed to 0.1 nmol L^−1^ AZA-2 was not significantly different from that of the control group. The strongest red fluorescence and the weakest green fluorescence appeared for Caco-2 cells exposed to 10 nmol L^−1^ of AZA-2. The results suggested that AZA-2 can induce apoptosis in Caco-2 cells in a dose-dependent manner.

### 2.2. Effect of AZA-2 on the Mitochondria of Caco-2 Cells

**Figure 3:**
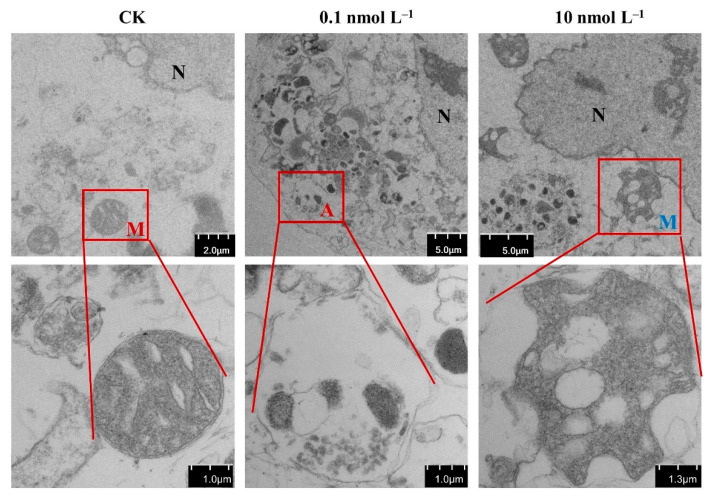
Effects of AZA-2 on the mitochondrial ultrastructure of Caco-2 cells monitored by a transmission electron microscope (TEM). Pictures in the second row indicate zooming in the area in the red box. “N” indicates the cell nucleus. “M” in red color indicates normal mitochondria; “M” in blue color indicates impaired mitochondria. “A” in red color indicates fusion of autophagosomes and lysosomes.

The mitochondrial ultrastructure of Caco-2 cells was observed in the control and AZA-2-exposed groups (Figure 3). Parallel cristae and the mitochondrial matrix were uniformly distributed in all mitochondria in the control group. In contrast, obvious mitochondrial swelling and cristae disorganization occurred in the AZA-2-exposed groups. Rupture of the mitochondrial membrane occurred and damaged mitochondria were translocated to lysosomes for cleavage in the highest concentration group of 10 nmol L^−1^. In addition, autophagic vacuoles encapsulating mitochondria were observed in cells exposed to 0.1 nmol L^−1^ of AZA-2, and more lysosomes were observed in the high concentration group, suggesting that AZA-2 induced the autolysis process in mitochondria. Excessive damage of organelles in Caco-2 cells can induce cell apoptosis. 

### 2.3. Oxidative Stress on Caco-2 Cells by AZA-2 Exposure

**Figure 4:**
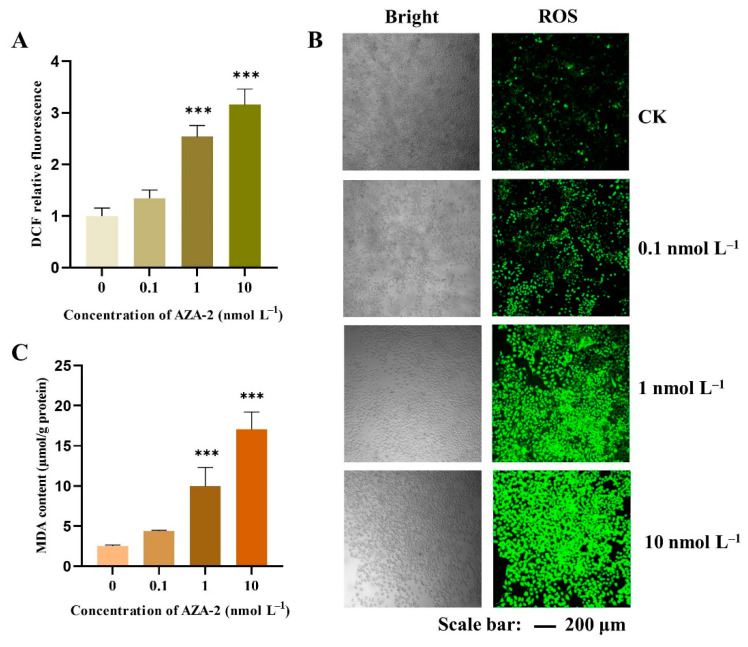
Relative fluorescence intensity (**A**) and laser scanning confocal microscope images (**B**) of ROS and the content of MDA (**C**) in Caco-2 cells exposed to different concentrations of AZA-2 for 48 h. Data are shown as mean values ± standard error (n = 3), and significant differences between AZA-2 exposed and control groups are indicated as *** *p* < 0.001.

The dichlorofluorescein fluorescence intensity of each AZA-2 exposure group relative to the control group is shown in Figure 4A. The exposure of AZA-2 induced ROS production in Caco-2 cells, where the ROS content in the 1 and 10 nmol L^−1^ exposure groups were 2.5- and 3.1-fold higher than that in the control group, respectively. No significant difference in ROS content was observed in the 0.1 nmol L^−1^ AZA-2 group compared with the control group. Green fluorescence was intensified with an increase in AZA-2 concentration in Caco-2 cells under confocal microscopy (Figure 4B), in which remarkable differences were found between the medium and high concentration groups and the control group. The results of the confocal microscopy photographs were consistent with the quantification of ROS fluorescence determined by flow cytometry.

The content of MDA was significantly increased in Caco-2 cells after 48 h of exposure to 1 and 10 nmol L^−1^ of AZA-2 (*p* < 0.001), with respective increases of 3.9- and 6.7-fold compared to the control group (Figure 4C). No significant difference in MDA content was recorded between the low concentration group and the control group. As the production of ROS continued to increase, the content of MDA also continued to increase in each group. As the exposure concentration of AZA-2 increased, the degree of lipid oxidation was higher in Caco-2 cells. 

### 2.4. Transcriptional Response to AZA-2 Exposure

**Figure 5:**
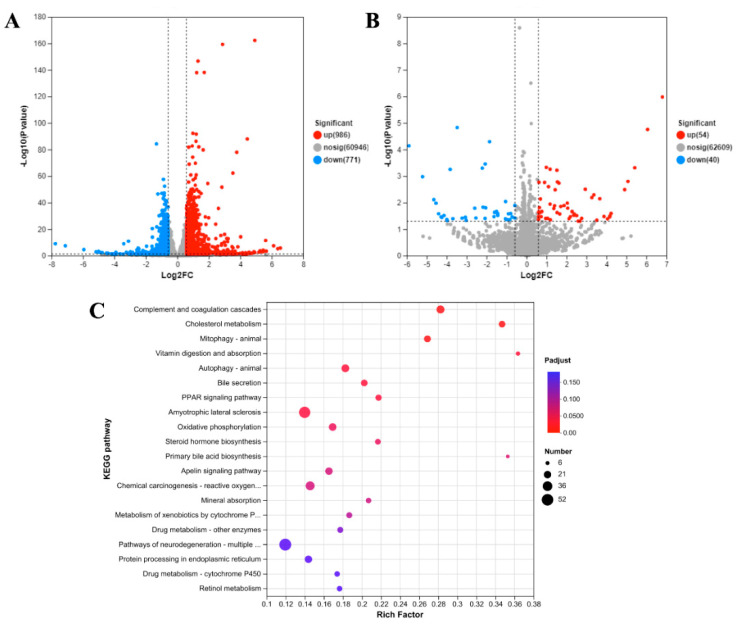
Volcano plot of the differential expressed genes (DEGs) of the Caco-2 cell line between 0.1 nmol L^−1^ (**A**) and 0.01 nmol L^−1^ (**B**) AZA-2 exposed groups and the control group, respectively, and the top 20 KEGG pathways enriched by DEGs (|fold change|> 2) in Caco-2 cells in the 0.1 nmol L^−1^ AZA-2 exposure and control groups (**C**).

Total RNA expression in Caco-2 cells exposed to AZA-2 at concentrations of 1/100 (0.1 nmol L^−1^) and 1/1000 EC_50_ (0.01 nmol L^−1^) for 48 h was analyzed using RNA-seq. A heatmap of differentially expressed genes (DEGs) and classification of the top 20 pathways enriched by KEGG are shown in Figure 5. Compared with the control group, 1757 DEGs were identified in the 0.1 nmol L^−1^ AZA-2-exposed group, of which 986 DEGs were up-regulated and 771 DEGs were down-regulated (Figure 5A), and 94 DEGs were identified in the 0.01 nmol L^−1^ AZA-2-exposed group, of which 54 DEGs were up-regulated and 40 DEGs were down-regulated (Figure 5B). A total of 323 KEGG pathways were enriched in all DEGs, of which 9 pathways were significantly expressed in the high-concentration group (Q value < 0.05), and no pathways were significantly changed in the 0.01 nmol L^−1^ AZA-2-exposed group (Figure 5C). These important KEGG pathways were mainly associated with cellular oxidative damage and lipid metabolism, including cholesterol metabolism, mitophagy, vitamin digestion and absorption, autophagy, bile secretion, the peroxisome proliferator-activated receptor (PPAR) signaling pathway and oxidative phosphorylation, while mitophagy and autophagy play important roles in scavenging in the oxidative damage system. No significant (Q value > 0.05) expression of the KEGG pathway was present in the 0.01 nmol L^−1^ AZA-2-exposed group.

## 3. Discussion

### 3.1. Cytotoxicity of AZA-2 on Caco-2 Cells

In this study, AZA-2 at concentrations of above 1 nmol L^−1^ markedly inhibited the proliferation of Caco-2 cells and led to cellular morphological changes. The 48 h EC_50_ of AZA-2 on Caco-2 cells was 12.65 nmol L^−1^ and was calculated by the neutral red method. Based on a volume of 500 mL fluid in the small intestine of a healthy adult [26], 400 g of shellfish per intake and a 47% leaching rate of AZA-2 from shellfish tissues during digestion in the human gastrointestinal tract [27], it is expected that the concentration of AZA-2 in the small intestine will be greater than 1 nmol L^−1^ when the concentration of AZA-2 in shellfish exceeds 2.28 µg kg^−1^. Previous studies have shown that concentrations of AZA-2 in some mollusks range from 0 to 9.96 µg kg^−1^ [8,9,10]. Therefore, consumption of AZA-2-contaminated seafood products may potentially harm human intestinal epithelial cells. 

In the present study, AZA-2 was found to markedly induce ROS production and lipid peroxidation in Caco-2 cells. Previous studies have found that the decrease in activities of respiratory chain complex I, III, IV (Cx I, Cx III, Cx IV) enzymes can inhibit oxidative phosphorylation and slow down electron transfer, thus substantially increasing ROS production [28]. Similar to complexes I and III, a decrease in cyclooxygenase (COX) activity further impedes electron transfer, leading to reduced COX function and ATP levels [29]. In this study, DEGs were essentially down-regulated in the oxidative phosphorylation pathway, where COX5B and COX7B gene expression were downregulated by 30% and 47%, respectively, which is consistent with the results of the effect of AZA-1 on T lymphocytes [17]. This suggests that inhibition of oxidative phosphorylation may be related to the production of ROS in the present study. In addition, transcriptomic data suggested that the accumulation of misfolded proteins occurred in the endoplasmic reticulum, which was also associated with the generation of ROS in cells [30]. The antioxidant defense cell system plays an important role in regulating cellular redox balance. The activities of antioxidant enzymes were not directly analyzed in this study, whereas transcriptome analysis showed that gene expression of nuclear factor kappa-B (NF-κB) and glutathione peroxidase (GPx) were up-regulated by 124% and 95%, respectively, which may play a role in scavenging ROS.

### 3.2. Autophagy of Caco-2 Cells Promoted by AZA-2

**Figure 6:**
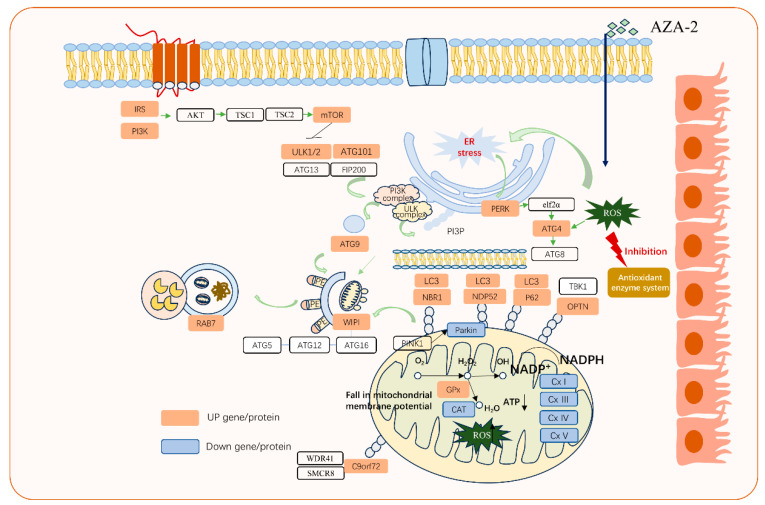
The autophagy and mitophagy KEGG pathways enriched by DEGs. Orange outline: up-regulation. Blue outline: down-regulation.

Autophagy is a cellular process of transporting cellular components to lysosomes or other vesicles for degradation [31]. The substrates of autophagy include aggregated proteins and damaged organelles such as mitochondria. The transmission electron microscopy results of this study showed that autophagic vesicles and damaged mitochondria occurred in Caco-2 cells exposed to 0.1 nmol L^−1^ of AZA-2 (Figure 3). Moreover, two important KEGG pathways, namely autophagy and mitophagy, were enriched by DEGs in the exposed Caco-2 cells in this study. Additionally, three endoplasmic reticulum sensors of inositol-requiring enzyme 1 (IRE1), protein kinase R-like endoplasmic reticulum (ER) kinase (PERK) and activating transcription factor 6 (ATF6) were significantly up-regulated by 46–89% in the present study and can activate the unfolded protein response to restore ER homeostasis [32]. The results suggest that misfolded proteins occurred in the endoplasmic reticulum in exposed Caco-2 cells, while AZA-2 induced cellular autophagy to remove damaged mitochondria and misfolded proteins (Figure 6).

Mitophagy is an important pathway to eliminate mitochondrial damage [33]. The first step involves activation of Parkin E3 ubiquitin ligase that marks damaged mitochondria [34]. Parkin is recruited by phosphatase and tensin homolog-induced putative kinase 1 (PINK1) to the ubiquitinated outer membrane of dysfunctional and depolarized mitochondria, where it associates with multiple proteins for recognition by autophagic machinery [35]. The autophagy receptor proteins of p62/sequestosome-1 (SQSTM1), neighbor of breast cancer gene 1 (NBR1), nuclear dot protein 52 (NDP52) and optineurin (OPTN) were up-regulated by 54–181% in exposed Caco-2 cells in this study. They act as signaling molecules to sequester mitochondria from the autophagosome, promoting the formation of autophagic lysosomes. Chromosome 9 open reading frame 72 (C9orf72) protein can promote the initiation of autophagy [36]. The complex of C9orf72 with Smith–Magenis syndrome chromosomal region candidate gene 8 (SMCR8) and WD40 repeat-containing protein 41 (WDR41) interacts with autophagy-related genes involved in autophagosome formation [37,38]. The increased expression of autophagy receptors and autophagy-initiating proteins promoted a 120% up-regulation of ubiquitin and autophagy component light chains 3 (LC3) expression in Caco-2 cells. Subsequently, LC3 interacted with autophagy receptors to transport ubiquitinated proteins to autophagosomes. 

In the autophagy pathway of the present study, the expression of unc-51-like kinases 1 (ULK1) was up-regulated by 53%. Once ULK1 is activated, autophosphorylation occurs, followed by phosphorylation of focal adhesion kinase family-interacting protein (FIP200) and autophagy-related protein13 (ATG13), driving the formation of an autophagosome. Two ubiquitin-like conjugation systems, namely ATG12 and LC3/ATG8 systems, are required for extension of the autophagosomal membrane [39]. A ubiquitin-like protein ATG12 initially forms a conjugate with ATG5 under the actions of ATG7 and ATG10. The ATG12–ATG5 conjugate then forms a complex with ATG16L1 [40]. Cytoplasmic LC3/ATG8 is converted to the LC3/ATG8–phosphatidylethanolamine (PE) conjugate by the proteases of ATG4, ATG7, ATG3 and the ATG12–ATG5–ATG16L1 complex [41]. In addition, the LC3/ATG8 protein associated with autophagosomes remains on the membrane of autophagosomes prior to fusion with lysosomes [42]. Upon binding with PE, LC3/ATG8 can recruit various proteins and facilitate cargo replenishment and transport, and lysosome fusion [43]. Once the autophagosome membrane is sealed, the autophagosome undergoes a maturation process during which it is transported to and fuses with lysosomes. 

### 3.3. Effects of AZA-2 on the Lipid Metabolism of Caco-2 Cells

In the present study, four important KEGG pathways related to lipid metabolism, i.e., cholesterol metabolism, vitamin digestion, bile secretion and the PPAR signaling pathway, were enriched by DEGs in exposed Caco-2 cells. Most DEGs involved in these pathways were down-regulated, suggesting that lipid metabolic processes were inhibited. 

ROS have been found to have a deleterious effect on lipids [44,45], and the failure to scavenge ROS in a timely manner can lead to the accumulation of free radicals, thus making lipids more susceptible to free radical attack and lipid peroxidation. The decrease in membrane fluidity due to lipid peroxidation can cause an increase in membrane leakage [46]. AZA-2 can inhibit vitamin C uptake by cyclic adenosine monophosphate (cAMP)-dependent transcriptional repression of sodium-dependent ascorbic acid membrane transporters solute carrier family 23 member 1 (SLC23A1) and SLC23A2 genes via the NF-κB signaling pathway [47]. In addition, vitamins, such as vitamin C, E and A, and polyphenols are antioxidant molecules. Vitamin C can donate electrons to prevent the oxidation of various ROS, reactive nitrogen species, sulfur radicals and nitrosylated compounds [48]. The inhibition of vitamin uptake in this study also suggests that the cellular antioxidant system is inhibited to some degree. Additionally, inhibition of COX5B in the oxidative phosphorylation pathway facilitated lipid accumulation by a reduction in fatty acid oxidation to protect against oxidative damage [49].

## 4. Conclusions

In summary, the results of this study showed that AZA-2 was cytotoxic to human intestinal epithelial Caco-2 cells in a concentration-dependent manner. The 48 h EC_50_ of AZA-2 on Caco-2 cells was 12.65 nmol L^−1^. Moreover, AZA-2 above 1 nmol L^−1^ induced substantial ROS production, lipid peroxidation and apoptosis in Caco-2 cells. Exposure to AZA-2 caused the formation of cellular autophagosomes, mitochondrial autophagy and cell lysis in Caco-2 cells. Transcriptional analysis revealed that most of the genes in the KEGG pathway associated with autophagy and mitochondrial autophagy were up-regulated, suggesting that the production of ROS induced by toxins triggered impairment of mitochondrial function and thus mitochondrial autophagy. Most of the genes in cholesterol metabolism, vitamin digestion, bile secretion and PPAR signaling pathways were down-regulated in the exposed Caco-2 cells, suggesting that lipid metabolic processes were inhibited. Overall, AZA-2 induced the production of large amounts of ROS in Caco-2 cells and caused oxidative damage, leading to mitochondrial damage and thus apoptosis and cell death.

## 5. Materials and Methods

### 5.1. Chemicals and Materials

Human intestinal epithelial Caco-2 cells were purchased from Qingqi Biotechnology Co., Ltd. (Shanghai, China). Assay kits of neutral red (NRU), malondialdehyde (MDA) and protein were obtained from Beyotime Biotechnology Co., Ltd. (Shanghai, China). The acridine orange/propidium iodide (AO/PI) staining kit was purchased from Beijing Solarbio Science & Technology Co., Ltd. (Beijing, China). The reactive oxygen species (ROS) assay kit was obtained from Nanjing Jiancheng Company (Nanjing, China). Purified AZA-2 (≥94% purity) was prepared by extraction from the microalga *Az. poporum* and purified successively using a Sephadex LH-20 chromatography column (1.6 cm × 100 cm), a semi-preparative liquid chromatograph (Hitachi, Primaide, Tokyo, Japan) and a hydrophile–lipophile balance solid phase extraction column (Oasis, 200 mg, 6 cc, Waters, Milford, CT, USA).

### 5.2. Cell Culture

The Caco-2 cell line was cultured in Dulbecco’s modified eagle medium (DMEM) with 10% fetal bovine serum, 100 U mL^−1^ penicillin and 100 μg mL^−1^ streptomycin in an incubator containing 5% CO_2_ at 37 °C. Caco-2 cells were seeded in 96-well or 6-well microplates at a density of approximately 5 × 10^4^ cells mL^−1^ and cultured for 48 h. The medium was then pipetted out and cells were washed twice with phosphate buffer solution (PBS). A methanol stock solution of AZA-2 was determined for concentration by LC-MS/MS and was then dried by nitrogen blow. The sample was reconstructed in DMEM medium containing 0.25% dimethyl sulfoxide (DMSO) to make a solution of AZA-2 at a concentration of 500 nmol L^−1^. The concentration of 500 nmol L^−1^ AZA-2 solution was then diluted stepwise to 300, 150, 50, 10, 5, 1, 0.5 and 0.1 nmol L^−1^ with DMEM medium containing 0.25% DMSO. Exposure concentrations of 0, 0.1, 0.5, 1, 5, 10, 50, 150, 300 and 500 nmol L^−1^ of AZA-2 were carried out to evaluate cell viability and those of 0, 0.1, 1 and 10 nmol L^−1^ were performed to investigate cellular apoptosis, ROS and MDA. Exposure concentrations of 0, 0.1 and 10 nmol L^−1^ were used to observe ultrastructural changes and those of 0, 0.01 and 0.1 nmol L^−1^ were performed to investigate transcriptome analysis. The control group was supplemented with only 0.25% DMSO. Each group was in triplicate. After 48 h of exposure, the medium was removed and the cells were washed once with PBS, and then the cells were used for subsequent assays. 

### 5.3. Cell Viability Assay

A volume of 20 µL neutral red solution and 200 µL DMEM medium were added to each well and incubated for 3 h at 37 °C. Cells were then rinsed twice with PBS, and 100 µL of solubilization solution (1% acetic acid in 50% ethanol) was added to each well, followed by blowing for 10 min to lyse the cells. Absorbance was read at 540 nm using a microplate reader (Thermo Multiskan Sky, Thermo Fisher Scientific, Waltham, MA, USA), and cell viability was expressed as optical density values relative to the control group. 

### 5.4. Acridine Orange/Propidium Iodide Staining

The apoptosis of Caco-2 cells was assessed by double staining with AO/PI. The exposed cells were stained with 100 μL of AO solution for 15 min; the supernatant was removed; and 200 μL of PI solution was added to stain for 5 min. Subsequently, the cells were washed twice with PBS, fixed with paraformaldehyde, and then observed by laser confocal microscopy (NIKONA1, NIKON, Tokyo, Japan).

### 5.5. Oxidative Stress Test

The accumulation of ROS in cells was determined by laser confocal microscopy and flow cytometry analysis. The rinsed cells were incubated with 200 μL of 10 μmol L^−1^ of the fluorescent probe 2′,7′-dichlorofluorescein diacetate (DCFH-DA) at 37 °C for 30 min in the dark. The DCFH-DA solution was then removed and the cells were washed twice with PBS. The cells were resuspended in 200 μL of PBS and observed by laser confocal microscopy (NIKONA1, NIKON, Tokyo, Japan). The obtained fluorescence images were processed using ImageJ software (v1.8.0.345) to count the fluorescence intensity of ROS.

In addition, the cells were digested with 1 mL of trypsin for 3 min and then mixed with 2 mL of DMEM medium to terminate the digestion. The cell mixture was centrifuged at 140× *g* for 3 min and the supernatant was decanted. The cells were resuspended in 400 μL of PBS. A volume of 450 μL of DCFA-DA was added to 50 μL of resuspended cells to make a final concentration of 10 µmol L^−1^. The sample was incubated for 30 min in the dark at 37 °C and centrifuged at 140× *g* for 3 min. The supernatant was decanted; the cell pellet was rinsed once with 300 μL of PBS; and then the cells were suspended in 300 μL of PBS. The fluorescence intensity of the resuspended cells was detected by an Accuri C6 plus Flow Cytometer (BD Biosciences, Franklin Lakes, NJ, USA).

The MDA and protein content were detected using assay kits. All assay procedures were performed according to kit instructions. A volume of 350 μL resuspended cells was sonicated for 1 min in an ice bath and then centrifuged at 140× *g* for 3 min at 4 °C. The supernatants were collected for MDA and protein content assays.

### 5.6. Observation by Transmission Electron Microscopy (TEM)

The morphological ultrastructure of the 0, 0.1 and 10 nmol L^−1^ AZA-2 exposure groups was observed using a transmission electron microscope (TEM, HT7800, Hitachi, Japan). The digestion and washing of the cells were the same as described above. The samples were fixed with 1% osmium solution for 2 h and washed three times with PBS. The samples were dehydrated stepwise with 30%, 50%, 70%, 80%, 90%, 95% and 100% ethanol solutions. Samples were sequentially treated with 100% acetone, 1:1 and 3:1 volume mixtures of embedding agent and acetone, and pure embedding agent. The embedded samples were cut into thin slices and stained sequentially with lead citrate, dioxane acetate and 50% ethanol. The samples were dried and observed for mitochondrial structure by transmission electron microscopy.

### 5.7. RNA Extraction, cDNA Library Construction and RNA-Sequencing Analysis 

Caco-2 cells were collected for transcriptome analysis with three samples per group after 48 h of exposure to 0, 0.01 and 0.1 nmol L^−1^ of AZA-2. The exposed cells were digested with 1 mL of trypsin and blown for 3 min. The digestion was then stopped by adding 5 mL of DMEM medium and blowing was continued for 5 min. The cell mixture was then centrifuged at 140× *g* for 3 min at 4 °C and the centrifugation step was repeated. The cells were then collected for total RNA extraction using TRIzol reagent following the manufacturer’s protocol. The quantity and quality of the total RNA were measured by an Agilent 5300 Bioanalyzer (Agilent Technologies, Palo Alto, CA, USA) and NanoDrop spectrophotometer ND-2000 (Thermo Fisher Scientific Inc.). The ranges of the RNA quality number (RQN) were 9.5–10.0 in all samples. RNA purification, reverse transcription, library construction and sequencing were performed at Shanghai Majorbio Bio-pharm Biotechnology Co., Ltd. (Shanghai, China). The expression level of each transcript was calculated to identify differential expression genes (DEGs) between two different samples. DESeq2 was used to detect DEGs, and DEGs with |log_2_FC| ≥ 1 and an adjusted *p* value (Padjust) < 0.05 were considered to be significantly differently expressed genes. In addition, KEGG (Kyoto Encyclopedia of Genes and Genomes) was used to identify which DEGs were significantly enriched in metabolic pathways at a *p*-value < 0.05 compared with the whole-transcriptome background. KEGG pathway analysis was carried out by Python Scipy software (v 1.4.1).

### 5.8. Statistical Analysis 

All data were expressed as mean ± standard deviation (n = 3). The value of EC_50_ was calculated by a probit analysis. One-way analysis of variance (ANOVA) followed by a least significant difference (LSD) test were employed to identify significant differences (α = 0.05) using IBM SPSS statistical package version 22. The figures were drawn by Origin 2019b and GraphPad prism 8. 

## Data Availability

All data can be supplied on request.

## Abbreviations

AO/PI	acridine orange/propidium iodide
ATF6	activating transcription factor 6
ATG	autophagy-related protein
AZA	azaspiracid
cAMP	cyclic adenosine monophosphate
cNDA	complementary deoxyribonucleic acid
COX	cyclooxygenase
C9orf72	chromosome 9 open reading frame 72
DCFH-DA	2′,7′-dichlorofluorescein diacetate
DEGs	differential expression genes
DMEM	Dulbecco’s modified eagle medium
DMSO	dimethyl sulfoxide
EC_50_	median effective concentration
ER	endoplasmic reticulum
FIP200	focal adhesion kinase family-interacting protein
GPx	glutathione peroxidase
IRE1	Inositol-requiring enzyme 1
KEGG	Kyoto encyclopedia of genes and genomes
LC3	light chains 3
MDA	malondialdehyde
NBR1	neighbor of breast cancer gene 1
NDP52	nuclear dot protein 52
NF-κB	nuclear factor kappa-B
NRU	neutral red
OPTN	optineurin
PBS	phosphate buffer solution
PCR	polymerase chain reaction
PE	phosphatidylethanolamine
PERK	protein kinase R-like endoplasmic reticulum kinase
PINK1	phosphatase and tensin homolog-induced putative kinase 1
PPAR	peroxisome proliferator-activated receptor
RNA-seq	ribonucleic acid sequences
ROS	reactive oxygen species
RQN	RNA quality number
SLC23A1	solute carrier family 23 member 1
SMCR8	Smith–Magenis syndrome chromosomal region candidate gene 8
SOD	superoxide dismutase
SQSTM1	sequestosome-1
ULK1	unc-51-like kinases 1
